# Arteriosclerosis, atherosclerosis, arteriolosclerosis, and Monckeberg medial calcific sclerosis: what is the difference?

**DOI:** 10.1590/1677-5449.200211

**Published:** 2021-06-25

**Authors:** Vanessa Prado dos Santos, Geanete Pozzan, Valter Castelli, Roberto Augusto Caffaro

**Affiliations:** 1 Universidade Federal da Bahia – UFBA, Salvador, BA, Brasil.; 2 Faculdade de Ciências Médicas da Santa Casa de São Paulo – FCMSCSP, São Paulo, SP, Brasil.

**Keywords:** atherosclerosis, arteriosclerosis, arteriolosclerosis, Monckeberg medial calcificsclerosis, vascular calcification

## Abstract

Cardiovascular diseases are the main cause of death in contemporary times. Arteriosclerosis, atherosclerosis, arteriolosclerosis, and Monckeberg's arteriosclerosis are terms that are often used interchangeably, but they refer to different vascular pathologies. The objective of this study is to review the concepts of atherosclerosis, atherosclerosis, arteriosclerosis and Monckeberg medial calcific sclerosis (MMCS). The term arteriosclerosis is more generic, meaning the stiffening and consequent loss of elasticity of the arterial wall, and encompasses the other terms. Atherosclerosis is an inflammatory disease secondary to lesions in the intimal layer and whose main complication is acute and chronic obstruction of the arterial lumen. Arteriolosclerosis refers to thickening of arterioles, particularly in association with systemic arterial hypertension. MMCS refers to non-obstructive calcification in the internal elastic lamina or the tunica media of muscular arteries. Vascular calcifications, which include atherosclerotic lesions and MMCS, have been studied as a risk factor for cardiovascular morbidity and mortality.

## INTRODUCTION

Cardiovascular diseases (CVD) are the main cause of morbidity and mortality worldwide.^1^ Globally, CVD were the cause of more than 17 million deaths in 2017.^1^ Ischemic cardiac disease was the number one cause of death from CVD, with around 8.93 million deaths, which, added to the 6.17 million deaths due to stroke, accounted for 84.9% of deaths from CVD worldwide.^1^

In Brazil, there were 424,058 deaths from CVD in 2015.^2^ Standardized for age, the mortality rate from CVD showed a 40.4% reduction from 1990 to 2015, falling from 429.5 per 100 thousand inhabitants to 256 per 100 thousand inhabitants.^2^ In the municipal district of São Paulo, a longitudinal study conducted from 2000 to 2010 showed an increasing prevalence of CVD among people over the age of 60 years, reaching 22.9%.^3^

In addition to heart diseases, disorders involving the arteries are also classified as CVD.^1,^ 2 Despite the reduction in mortality due to ischemic heart disease and cerebrovascular disease among Brazilians from 1990 to 2015, there was an increase in mortality of both sexes due to peripheral vascular disease (PVD), increasing from 0.9 to 1.6 per 100 thousand inhabitants, which equates to an 82.1% increase.^2^

The arteries have attracted medical and scientific interest for a very long time. Around 200 years BC, Galen conducted an experiment demonstrating that the arteries contained blood, not air.^4^ The arteries are classified into elastic and muscular arteries, according to their composition, and with areas of transition between the two different types.^5^ The artery wall has three layers, or tunicae.^5^ The most inward layer is the tunica intima, which is lined with endothelial cells. The next layer is the tunica media, which contains smooth muscle cells and, the outer layer is the tunica adventitia, made up of connective tissues, collagen, and elastic fibers.^5^ The intima and media are separated by the internal elastic lamina, whereas the external elastic lamina delineates the division between the medial and adventitial layers.^5^

Many diseases course with artery wall disorders. These include arteriosclerosis, atherosclerosis (AT), arteriolosclerosis, and Monckeberg medial calcific sclerosis (MMCS), which are terms used by healthcare professionals in their daily routines and found in the literature. The four terms are sometimes treated as synonyms, but they describe different concepts, with distinct morphological aspects.^6^^,^^7^

The objective of this study was to review the concepts of arteriosclerosis, AT, arteriolosclerosis, and MMCS, which are found in the literature and used by healthcare professionals in their daily practice. A narrative literature review was conducted, seeking references that would contribute to understanding of these concepts. Randomized clinical trials and observational studies were classified with their evidence levels in parentheses.^8^

## ARTERIOSCLEROSIS

Arteriosclerosis is a word with Greek origins that means hardening or stiffening of the artery wall.^7^ The term arteriosclerosis tends to be employed generically, including three different disorders: AT, arteriolosclerosis, and MMCS.^7^

Hardening of the vascular walls, known as arteriosclerosis, can lead to increased systolic and pulse pressure, with consequent ventricular hypertrophy, which is one of the factors associated with CVD mortality.^6^ The type of vessel predominantly involved in each of the pathological processes can also differ. Whereas AT involves large and medium caliber arteries, the term arteriolosclerosis denotes compromised arterioles.^7^

## ATHEROSCLEROSIS

Atherosclerosis is considered a chronic inflammatory disease, with involvement of both innate and adaptive immunity and participation of macrophages and lymphocytes in the atherosclerotic process.^9^^,^^10^ In the context of this definition, the role of inflammatory biomarkers as a risk factor for cardiovascular events and monitoring treatment has been studied in the literature.^9^ A randomized, controlled, and double-blind study (the JUPITER trial) showed that there was a reduction in high-sensitivity C-reactive protein levels among patients who used the medication Rosuvastatin, with a reduction in cardiovascular events (level 1b).^11^

Risk factors associated with AT are subdivided into environmental factors and genetic conditions.^12^ Smoking, a diet rich in lipids and inactivity are environmental factors that can be modified, whereas sex, diabetes mellitus (DM), systemic arterial hypertension (SAH), and family history have strong genetic components.^12^ Age, DM, SAH, dyslipidemia, and smoking are factors associated with increased risk of CVD and PVD, and the main cause of these diseases is AT.^3^^,^^10^

Advanced age contributes to increased prevalence of CVD (level 2b).^3^ Among the elderly, increasing age is associated with higher prevalence of CVD and, as the life expectancy of the Brazilian population increases (76.3 years overall and 72.8 years for men and 79.9 years for women), this risk factor has a growing impact.^3^^,^^13^ Another important risk factor, DM has an estimated prevalence in Brazil of 6.6 to 9.4% of the population and rates are higher among older people, women, persons with low educational level, and people with overweight and obesity (level 2b).^14^ Diabetes Mellitus is considered a risk factor for AT, heart failure, intermittent claudication, and mortality from CVD in both sexes.^15^ With regard to risk factors for CVD, although smoking rates have fallen, upward trend in rates of overweight and obesity, physical inactivity, and DM have been observed among Brazilians.^16^

The pathogenesis of atherosclerotic disease is linked to injury or dysfunction of endothelial cells, determined by the many different risk factors, modifiable or non-modifiable, creating a proinflammatory and pro-thrombotic environment, consisting of increased vascular permeability, influx of lipids (cholesterol and esters of cholesterol), adhesion of blood monocytes and lymphocytes, adhesion of platelets, and expression of growth factors with proliferation of smooth muscle fibers, which migrate to the intima, where they produce extracellular matrix.^9^^,^^17^ The atherosclerotic plaques that are formed in this manner have a lipid nucleus, formed by cholesterol and esters of cholesterol present in activated macrophages and smooth muscle cells (xanthomatous cells) or in the extracellular space in the form of cholesterol crystals, lymphocytes, and necrotic remnants, in addition to a fibrous cap formed from collagen produced by the smooth muscle fibers that have migrated to the intima. The variable proportion of these elements determines different morphological aspects which, in turn, are related to different clinical presentations and are associated with varying cardiovascular risks.^18^

One histological classification for atherosclerotic lesions employs a numerical grade based on the characteristics of the plaque.^19^ Initial lesions, types I, II, and III, tend to be small and clinically silent and have little or no disorganization of the tunica intima.^17^ Type I lesions have accumulation of intracellular lipids with isolated foam cells; type II lesions have greater numbers of foam cells, with fatty streaks; and type III lesions, known as intermediate or transition lesions, have foam cells and small extracellular accumulations of lipids.^17^ Type III lesions are also known as pre-atheroma, and are found in young adults.^17^

**Figure 1:**
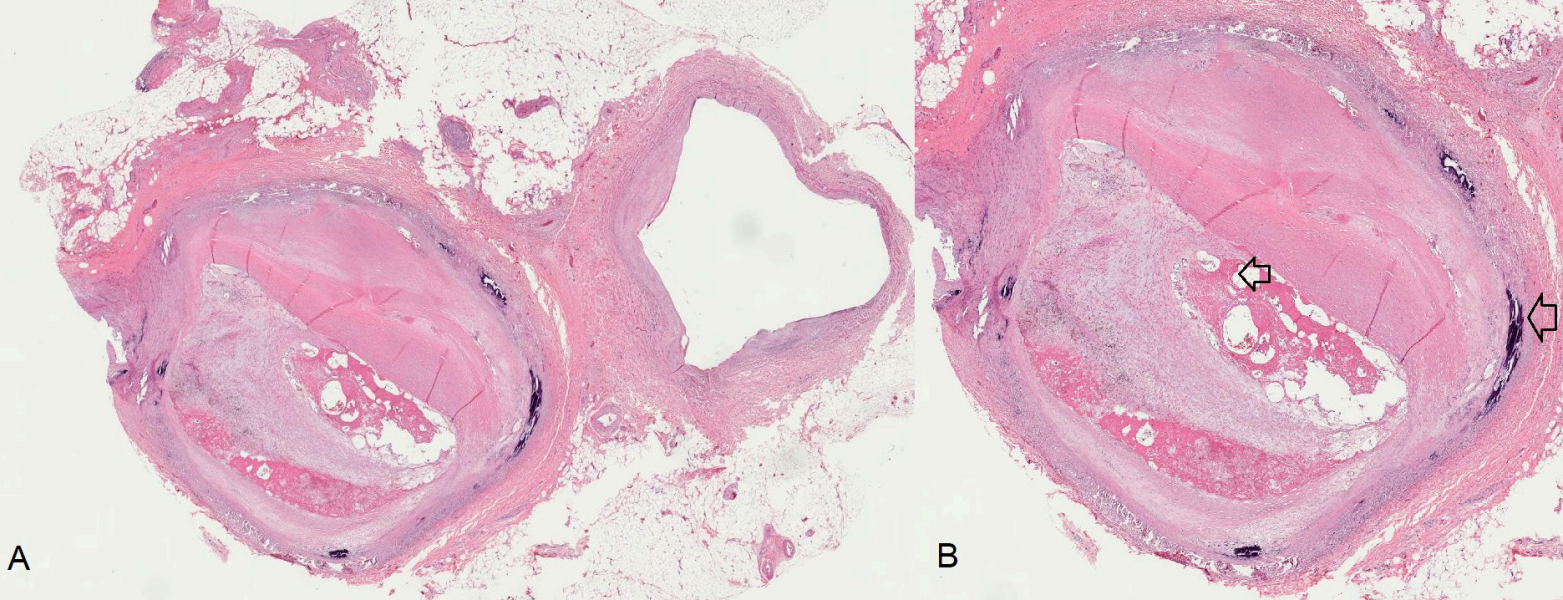
Histological section of peripheral vascular bundle from a lower limb with atherosclerotic lesion: **A)** Peripheral vascular bundle with total obstruction of the arterial lumen. **B)** In the detailed image, Monckeberg medial calcific sclerosis can be observed in the periphery (external arrow), while occlusive arterial thrombosis in organization is seen centrally (internal arrow).

**Figure 2:**
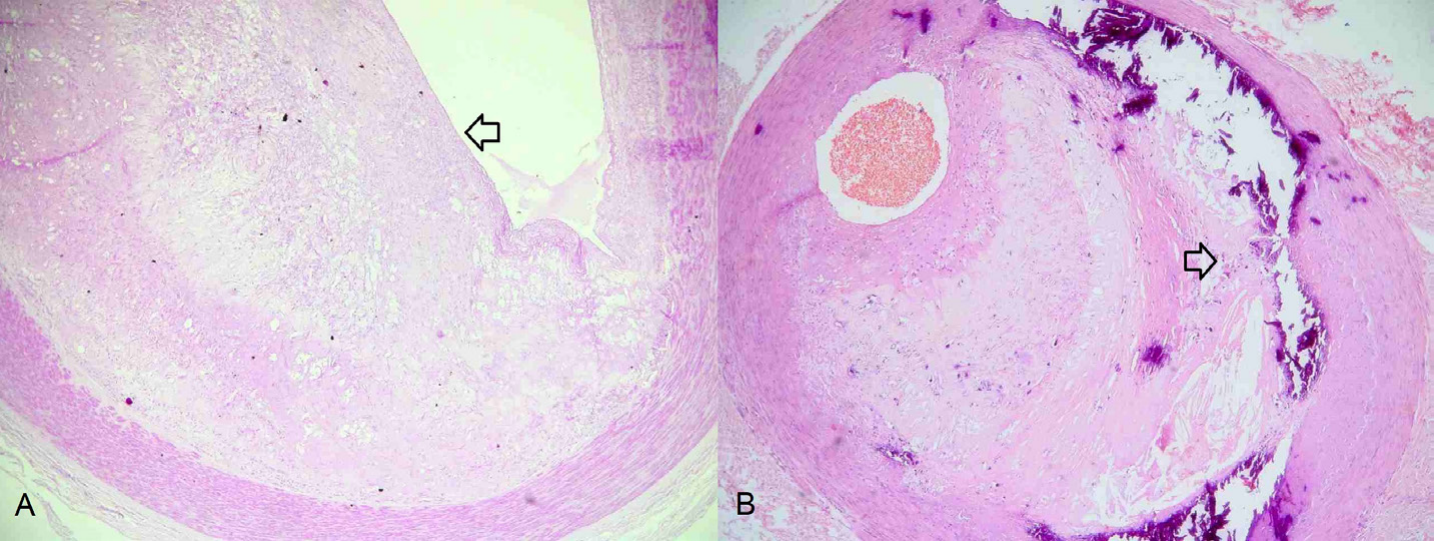
Histological sections of peripheral arterial segments from lower limbs with atherosclerotic lesions. **A)** Atherosclerotic lesion of the fibroatheroma type (arrow). **B)** Complicated atherosclerotic lesion with presence of calcification of the plaque (arrow).

Advanced lesions start with type IV, or atheroma, in which there is a lipid core that is well-formed with foam cells and extracellular deposits in the form of cholesterol crystals.^17^ Type V lesions, or fibroatheroma, are characterized by a well-developed lipid core covered by a fibrous capsule. Type VI lesions are known as complicated lesions (Figure 1), because of presence of disorders such as intraplaque hemorrhage, fissures, erosions, or thrombosis.^19^ An update to this classification provided greater detail on complicated lesions (Figure 2), adding type VII atherosclerotic lesions, in which calcification predominates, and type VIII lesions, in which fibrosis predominates.^20^

The progression of lesions does not necessarily occur in a single direction or in a strictly sequential manner.^20^ Atheromas, or advanced type IV lesions, can develop directly to types V, VI, VII, or VIII, without necessarily proceeding through each of those stages.^20^ Other classifications have been proposed in the literature, also based on atherosclerotic lesions in coronary arteries, but considering presence of erosion and plaque rupture and also presence of thrombi.^18^

The morphology of atherosclerotic plaques plays an important role in clinical presentation. Whereas clinically evident chronic obstructive lesions are generally fibroatheromas, in which the lipid core is small and the fibrous cap is well developed, making the artery more rigid, acute obstructions due to thrombosis more frequently form on plaques with a large lipid core and a thin or inexistent fibrous cap, known as thin-cap fibroatheromas, soft plaques, or vulnerable plaques.^21^ The well-developed lipid core is indicative of predominance of the proinflammatory environment, with large numbers of activated macrophages secreting metalloproteinases that lyse the fibrous cap and make the plaque more predisposed to fissures and ulcerations.^9^

Clinical presentation can vary depending on the degree of lumen obstruction and the velocity with which the obstruction develops. Chronic obstruction can become clinically evident when the lumen is reduced by at least 70%. However, acute obstructions, associated with thrombosis, can occur with clinically silent plaques.

In the peripheral arteries, a study of patients with peripheral arterial occlusive disease (PAOD) showed that in patients who had undergone an amputation there were advanced atherosclerotic lesions in arterial segments, the majority of which were types V and VI, with degree of obstruction exceeding 75% (level 3b).^22^ Another study including cases with critical limb ischemia, and analyzing arteries of amputated lower limbs, found a predominance of fibroatheroma lesions and plaques with fibrocalcification in the femoropopliteal segment, and an elevated frequency of thrombi and calcification of the tunica media in infrapopliteal arteries (level 3b).^23^

## ARTERIOLOSCLEROSIS

Arterioles are part of the microvascular bed, located between the terminal arteries and the capillaries, and are responsible for the greater part of peripheral resistance to blood flow.^24^ The terminal arteries and the arterioles control resistance to blood flow, contributing to control of arterial blood pressure and regulating tissue perfusion.^24^ The arteriole wall has cellular and extracellular components, the greater part of which are composed of smooth muscle cells, which control the diameter of the vessel, responding with vasoconstriction or vasodilation to the various different physiological stimuli.^5^^,^^24^

Arteriolosclerosis is thickening of the arteriole walls. It can be present in many different tissues and organs and is observed in a variety of diseases. Thickening of the arterioles has been described among the histological findings of liver disease secondary to schistosomiasis.^25^ Systemic arterial hypertension is one of the risk factors implicated in AT and is also related to arteriolosclerosis.^26^^,^^27^

There are two types of arteriole thickening: hyaline arteriolosclerosis and hyperplastic arteriolosclerosis.^7^ Hyaline arteriolosclerosis is frequently associated with SAH (level 2b).^26^^-^28 The kidney and brain are both organs that can be compromised by arteriolosclerosis.^26^^,^^29^ In the kidneys of hypertensive patients, hyaline arteriolosclerosis of the afferent glomerular arteriole causes chronic ischemia and consequent glomerulosclerosis, responsible for progression to chronic renal failure.^30^ In the brain, hyaline arteriolosclerosis and formation of Charcot-Bouchard microaneurysms are responsible for intraparenchymal hemorrhage, an important complication in hypertense patients. Hyperplastic arteriolosclerosis is characterized by laminar and concentric thickening, which cause significant reduction of the arteriole lumen, consist of smooth muscle cells with thickened and multiple basement membranes, and are associated with severe SAH cases.^30^

Mechanisms that regulate the activities of arterioles at the molecular level have been studied in experimental animal models.^31^ A study that was conducted with data from patients with Alzheimer’s disease found that cerebral arteriolosclerosis was associated with cognitive changes in the elderly (level 2b).^29^ In the lower limbs of patients with advanced PAOD, another study found arteriole thickening in half of non-diabetic patients and 63% of diabetic patients, with no significant difference between the groups (level 3b).^22^

## MONCKEBERG MEDIAL CALCIFIC SCLEROSIS

**Figure 3:**
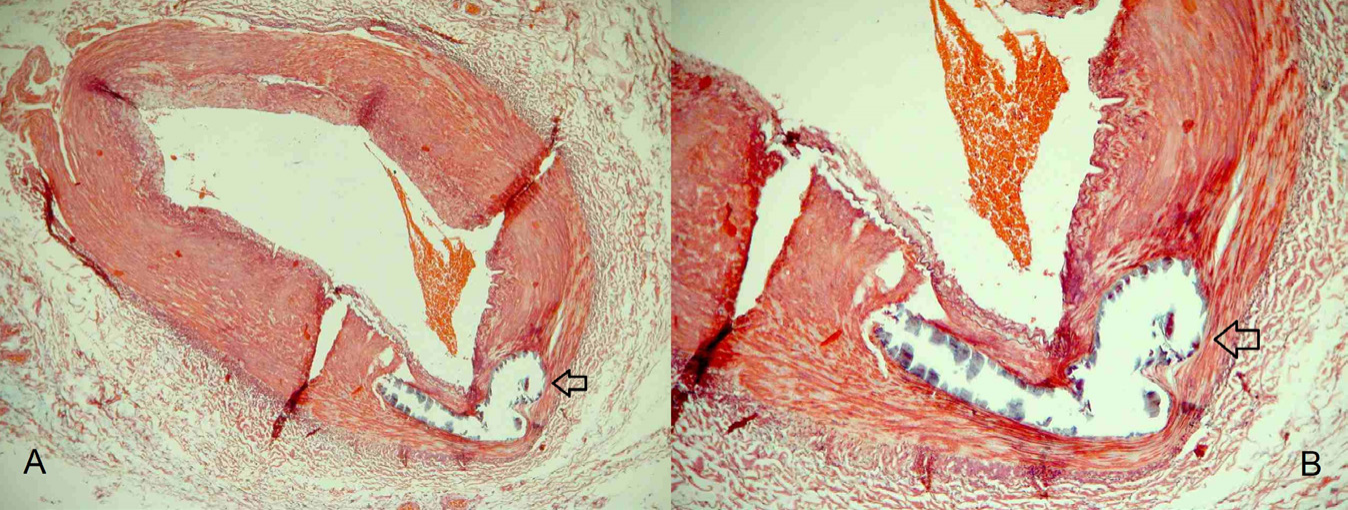
Histological section of a peripheral arterial segment from the lower limb, showing calcification located in the arterial tunica media, or Monckeberg medial calcific sclerosis (arrows).

Monckeberg medial calcific sclerosis is also known by other denominations , such as medial arterial calcification (MAC), Monckeberg’s arteriosclerosis, and Monckeberg medial calcinosis^6^^,^^32^ (Figure 3). It was first described in 1903 as a calcification located in the middle layer of the walls of arteries that does not involve the intima.^32^^-^34 Considering the original description, there are disagreements in the literature on whether or not MMCS includes calcifications located in the internal elastic lamina.^7^^,^^32^ An observational study suggested that calcification in MMCS could be present both in the arterial tunica media and in the internal elastic lamina (level 4).^33^ Some authors have proposed changing the classification and nomenclature of artery wall lesions to make the characteristics of each lesion clearer.^7^

Different mechanisms may be implicated in MMCS, such as activation and migration of myofibroblasts from the adventitia and differentiation of smooth muscle cells.^6^ The distinct clinical conditions associated with MMCS include DM, chronic kidney disease (CKD), and advanced age.^6^^,^^32^^,^^35^^,^^36^ In general, MMCS is rare before 50 years of age, but it may have onset earlier in CKD, even in the absence of atherosclerotic lesions.^7^^,^^32^ Monckeberg medial calcific sclerosis is considered a type of vascular calcification (VC) and has been associated with increased cardiovascular morbidity and mortality.^6^^,^^35^ A cohort study that enrolled adults with no history of CVD suggested a possible association between an elevated ankle-brachial index (ABI) (≥ 1.4) and increased risk of cardiovascular mortality (level 2b).^37^

**Figure 4:**
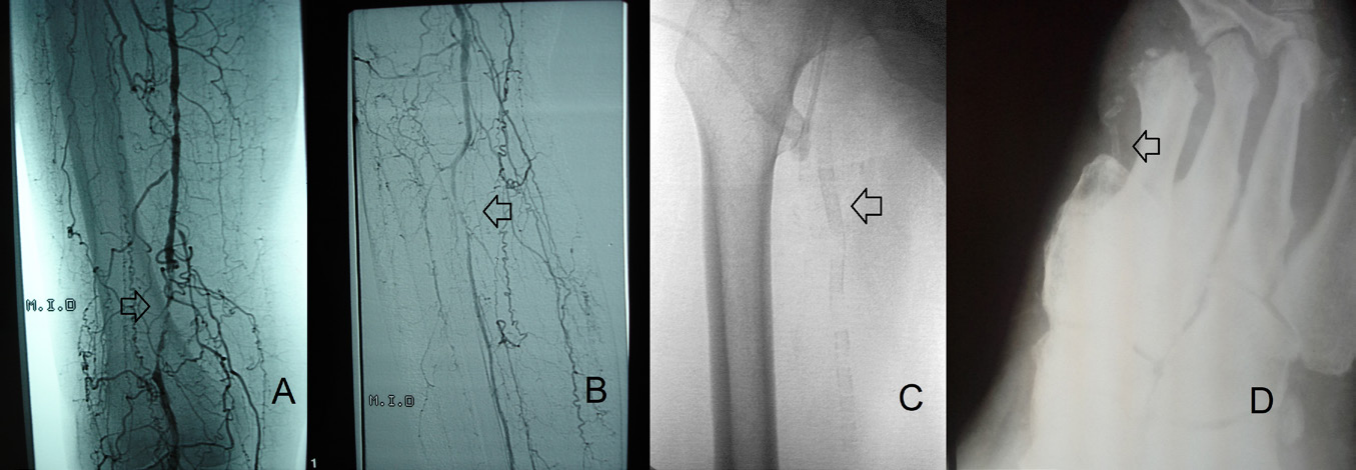
**A & B)** Digital arteriography with contrast showing images of arterial obstructions compatible with atherosclerosis (arrows). **C & D)** Radioscopy and X-ray without contrast showing images compatible with Monckeberg medial calcific sclerosis (arrows).

The ABI is a noninvasive diagnostic method that is suggestive of presence of MMCS when found to be abnormal, although it can be falsely elevated in diabetic patients with lower limb ischemia.^35^^,^^36^^,^^38^ Calcification of the tunica media of distal arteries (Figure 4) can interfere with compression by the sphygmomanometer, resulting in a falsely elevated ABI value. An observational study found that 11% of diabetic patients with a diagnosis of critical ischemia had a falsely elevated ABI (level 3b).^38^

Classically, MMCS does not manifest as an obstructive lesion and does not compromise the arterial lumen, but it can coexist with atherosclerotic plaques.^7^^,^^39^ Even MMCS does not have an obstructive nature, in contrast with advanced atherosclerotic lesions, both types of VC can cause intraoperative difficulties during arterial surgery.^35^

## VASCULAR CALCIFICATION

The term VC is employed in the literature to refer to a set of conditions characterized by ectopic calcification in vascular territories.^6^ The four distinct disorders included in the class of vascular calcifications are MMCS, calcification in the atherosclerotic plaque, calcification of cardiac valves, and calciphylaxis.^6^^,^^32^ Of the diseases discussed here, calcification may occur in advanced atherosclerotic lesions and in MMCS, and it can be difficult to distinguish the exact site of calcification in tomographic images, whether the arterial intima or media.^7^^,^^35^^,^^39^

Computed tomography is an imaging method that is used to formulate VC scores in many different arterial, coronary, cerebral, and peripheral territories.^40^^-^42 A prospective multicenter study that evaluated the accuracy of 64 channel multislice computed tomography angiography for diagnosis of coronary stenosis found sensitivity of 95% for stenosis ≥ 50% and 94% for stenosis ≥ 70% (level 1c).^43^

In the intracranial arteries, a cohort study revealed an association between tomographic calcification scores, occurrence of vascular, coronary, cerebral, or peripheral events, and deaths of patients who had had an ischemic stroke (level 2b).^42^ A multicenter cohort study showed that individuals with higher calcium measurements in the coronary arteries exhibited higher risk of cardiovascular events due to atherosclerotic disease in the different territories, regardless of age, sex, and ethnicity (level 2b).^44^

In the peripheral arteries, VC scores are also being proposed for patients with PVD of the lower limbs.^35^^,^^40^ A retrospective observational study, that enrolled patients with symptomatic PVD, showed that diabetics and people with CKD had higher calcification scores and that tomographic peripheral artery calcification scores were associated with greater cardiovascular morbidity and mortality (level 2c).^40^

Both, MMCS and calcified atherosclerotic lesions, have risk factors in common, such as advanced age and DM, and they can occur in isolation or they can coexist in the artery wall.^39^ Both types of VC can cause problems for access, catheterization, angioplasty, re-entry techniques, clamping, and anastomosis, in both conventional surgery and endovascular procedures. In infrainguinal arteries, VC makes recanalization attempts challenging, increasing the risk of perforation, dissection, and distal embolization.^35^ The literature contains studies seeking treatment options for the different forms of VC, whether investigating means of prevention of calcification or proposing new devices capable of treating calcifed arteries with better results.^35^^,^^45^^,^^46^

## CONCLUSIONS

The medical practice uses similar terms to describe distinct arterial diseases, which can make understanding difficult. This review discussed the concepts involved in these different disorders.

Whereas the term arteriosclerosis encompasses all lesions that lead to hardening of the arteries, atherosclerosis refers to presence of atheromatous plaque, with lipids accumulate in the in the arterial tunica intima. In turn, arteriolosclerosis describes cellular or hyaline thickening of microvascular bed vessels, the arterioles. Finally, MMCS is the presence of calcification in the internal elastic lamina or in the middle layer of muscular arteries. Atherosclerotic lesions and MMCS are included among the vascular calcifications, which are studied as risk factor for cardiovascular events.
