# Supplementary material for: Arteriosclerosis, atherosclerosis, arteriolosclerosis, and Monckeberg medial calcific sclerosis: what is the difference?
Source: J Vasc Bras. 2021 Jun 25;20:e20200211. doi: 10.1590/1677-5449.200211 (PMC8276643; doi:10.1590/1677-5449.200211)
Supplement: Translation

# Translation: Arteriosclerose, aterosclerose, arteriolosclerose e esclerose calcificante da média de Monckeberg: qual a diferença?

**Authors:** Vanessa Prado dos Santos, Geanete Pozzan, Valter Castelli, Roberto Augusto Caffaro

**Affiliations:** 1 Universidade Federal da Bahia – UFBA, Salvador, BA, Brasil.; 2 Faculdade de Ciências Médicas da Santa Casa de São Paulo – FCMSCSP, São Paulo, SP, Brasil.

**Keywords:** aterosclerose, arteriosclerose, arteriolosclerose, esclerose calcificante da média de Monckeberg, calcificação vascular

## Abstract

A principal causa de óbito na contemporaneidade são as doenças cardiovasculares. Arteriosclerose, aterosclerose, arteriolosclerose e arteriosclerose de Monckeberg são termos frequentemente utilizados como sinônimos, mas traduzem alterações distintas. O objetivo desta revisão foi discutir os conceitos de arteriosclerose, aterosclerose, arteriolosclerose e esclerose calcificante da média de Monckeberg. O termo arteriosclerose é considerado mais genérico, significando o enrijecimento e a consequente perda de elasticidade da parede arterial, abarcando os demais tipos. A aterosclerose é uma doença inflamatória secundária a lesões na camada íntima, que tem como principal complicação obstrução crônica e aguda do lúmen arterial. A arteriolosclerose se refere ao espessamento das arteríolas, particularmente relacionada à hipertensão arterial sistêmica. Já a esclerose calcificante da média de Monckeberg designa a calcificação, não obstrutiva, da lâmina elástica interna ou da túnica média de artérias musculares. As calcificações vasculares, que incluem lesões ateroscleróticas e a esclerose calcificante da média de Monckeberg, vêm sendo estudadas como um fator de risco para a morbimortalidade cardiovascular.

## INTRODUÇÃO

As doenças cardiovasculares (DCV) são uma causa importante de morbimortalidade no mundo^1^. Globalmente, as DCV causaram mais de 17 milhões de mortes em 2017^1^. A doença cardíaca isquêmica foi a principal causa de morte por DCV, com cerca de 8,93 milhões de óbitos, os quais, somados aos 6,17 milhões de mortes por acidente vascular encefálico (AVE), representaram 84,9% dos óbitos por DCV em todo o mundo^1^.

No Brasil, ocorreram 424.058 óbitos por DCV em 2015^2^. A taxa de mortalidade por DCV, padronizada por idade, apresentou um decréscimo de 40,4% entre 1990 e 2015, migrando de 429,5 por 100 mil habitantes para 256 a cada 100 mil habitantes^2^. No município de São Paulo, um estudo longitudinal, feito entre 2000 e 2010, revelou uma prevalência crescente de DCV entre os indivíduos com mais de 60 anos, chegando a 22,9%^3^.

Além das cardiopatias, as doenças que acometem as artérias também estão classificadas entre as DCV^1,^ 2. Apesar da diminuição da mortalidade por cardiopatia isquêmica e doença cerebrovascular entre os brasileiros, de 1990 a 2015, houve uma elevação da mortalidade por doença vascular periférica (DVP) em ambos os sexos, indo de 0,9 para 1,6, a cada 100 mil habitantes, o que representa um aumento de 82,1%^2^.

As artérias despertam interesse médico e científico ao longo dos tempos. Cerca de 200 anos d.C., Galeno demonstrou, através de um experimento, que as artérias continham sangue, e não ar^4^. A classificação das artérias subdivide-as em artérias elásticas e musculares, de acordo com a sua composição, havendo áreas de transição entre os diferentes tipos^5^. A parede arterial é composta por três camadas ou túnicas^5^. A mais interna é a íntima, recoberta por células endoteliais, seguida da camada média, que contém as células musculares lisas e, mais externamente, a túnica adventícia, formada por tecido conjuntivo, colágeno e fibras elásticas^5^. O limite entre as túnicas, a íntima e a média é demarcado pela lâmina elástica interna, enquanto a lâmina elástica externa marca a distinção entre as camadas média e adventícia^5^.

Diversas doenças cursam com o comprometimento da parede arterial. Entre elas, estão as que denominamos arteriosclerose, aterosclerose (AT), arteriolosclerose e esclerose calcificante da média de Monckeberg (ECMM), termos empregados no cotidiano das profissões da área da saúde e na literatura. Por vezes utilizados como sinônimos, os quatro termos traduzem conceitos diferentes, com aspectos morfológicos distintos^6^^,^^7^.

O objetivo deste estudo foi revisar os conceitos de arteriosclerose, AT, arteriolosclerose e ECMM, que são termos comumente encontrados na literatura especializada e utilizados no cotidiano dos profissionais de saúde. Foi realizada uma revisão narrativa da literatura, buscando referências que contribuíssem para a compreensão dos conceitos. Os ensaios clínicos randomizados e os estudos observacionais foram classificados de acordo com seu nível de evidência entre parênteses^8^.

## ARTERIOSCLEROSE

Arteriosclerose é uma palavra de origem grega, que significa o endurecimento ou o enrijecimento da parede arterial^7^. O termo arteriosclerose costuma ser empregado de maneira genérica, incluindo estas três moléstias diferentes: a AT, a arteriolosclerose e a ECMM^7^.

O enrijecimento da parede vascular, chamado de arteriosclerose, pode levar ao aumento da pressão sistólica e da pressão de pulso, com consequente hipertrofia ventricular, um dos fatores associados à mortalidade por DCV^6^. O tipo de vaso predominantemente envolvido em cada um desses processos patológicos também pode diferir. Enquanto a AT acomete as artérias de grande e médio calibre, o termo arteriolosclerose denota o comprometimento das arteríolas^7^.

## ATEROSCLEROSE

A ateroscleose (AT) vem sendo considerada uma doença inflamatória crônica, com atuação tanto da imunidade inata quanto adaptativa, e participação de macrófagos e linfócitos no processo aterosclerótico^9^^,^^10^. No contexto dessa definição, o papel dos biomarcadores inflamatórios na avaliação do risco de eventos cardiovasculares e no acompanhamento terapêutico vem sendo estudado na literatura^9^. Um estudo randomizado, controlado e duplo-cego (JUPITER *Trial*) mostrou que houve uma diminuição dos níveis de proteína C reativa de alta sensibilidade nos pacientes que utilizaram a medicação Rosuvastatina, com redução de eventos cardiovasculares (nível 1b)^11^.

Os fatores de risco associados à AT estão subdivididos em fatores ambientais e condições genéticas^12^. Tabagismo, dieta rica em lipídios e sedentarismo são fatores ambientais e modificáveis, enquanto gênero, diabetes melito (DM), hipertensão arterial sistêmica (HAS) e história familiar possuem um forte componente genético^12^. Idade, DM, HAS, dislipidemia e tabagismo são fatores associados ao maior risco de DCV e DVP, que têm como principal causa a AT^3^^,^^10^.

A idade avançada contribui para uma maior prevalência de DCV (nível 2b)^3^. Entre idosos, o aumento da faixa etária está associado a uma maior prevalência de DCV, e, com a crescente expectativa de vida da população brasileira — 76,3 anos no geral, sendo 72,8 anos para os homens e 79,9 anos para as mulheres —, percebe-se o papel de destaque desse fator de risco^3^^,^^13^. Outro importante fator de risco, o DM, tem uma prevalência estimada no país de 6,6 a 9,4% da população, sendo maior quanto maior a idade, entre as mulheres, entre pessoas com baixa escolaridade e entre indivíduos com sobrepeso e obesidade (nível 2b)^14^. O DM é considerado um fator de risco para AT, insuficiência cardíaca, claudicação intermitente e mortalidade por DCV em ambos os sexos^15^. Entre os fatores de risco de DCV, mesmo tendo havido uma diminuição do tabagismo, tem sido observada uma tendência de aumento dos índices de sobrepeso e obesidade, inatividade física e DM entre os brasileiros^16^.

A patogenia da doença aterosclerótica está relacionada a lesão ou disfunção da célula endotelial, determinada pelos diversos fatores de risco, modificáveis ou não, criando um ambiente pró-inflamatório e pró-trombótico, representado por aumento da permeabilidade vascular, entrada de lípides (colesterol e ésteres de colesterol), adesão de monócitos sanguíneos e linfócitos, adesão de plaquetas e expressão de fatores de crescimento com proliferação das fibras musculares lisas e sua migração para a íntima, onde produzem matriz extracelular^9^^,^^17^. A placa aterosclerótica assim formada terá um núcleo lipídico, formado por colesterol e ésteres de colesterol presentes em macrófagos ativados e células musculares lisas (células xantomizadas) ou no extracelular sob a forma de cristais de colesterol, linfócitos e restos necróticos, além de uma capa fibrosa formada por colágeno produzido pelas fibras musculares lisas que migraram para a íntima. A variável proporção desses elementos determina diferentes aspectos morfológicos que, por sua vez, estão relacionados a diferentes comportamentos clínicos e associados a risco cardiovascular variável^18^.

Uma das classificações histológicas propostas para as lesões ateroscleróticas utiliza uma divisão numérica, de acordo com as características da placa^19^. As lesões iniciais, dos tipos I, II e III, costumam ser pequenas e clinicamente silenciosas, e há pouca ou nenhuma desorganização da túnica íntima^17^. Na lesão tipo I, existe o acúmulo de lipídeos intracelulares com células espumosas isoladas; na lesão tipo II, há o maior acúmulo de células espumosas com estrias lipídicas; e na lesão tipo III, chamada de lesão intermediária ou de transição, existem células espumosas e pequenos acúmulos extracelulares de lipídeos^17^. A lesão tipo III também é denominada pré-ateroma, sendo encontrada em adultos jovens^17^.

As lesões avançadas se iniciam com o tipo IV ou ateroma, em que se encontra o núcleo lipídico, ou *core* lipídico, bem formado com células espumosas e depósitos extracelulares sob a forma de cristais de colesterol^17^. A lesão tipo V, ou fibroateroma, se caracteriza pelo núcleo lipídico bem desenvolvido, recoberto pela cápsula fibrosa. A lesão tipo VI é chamada de lesão complicada (Figura 1), devido à presença de alterações como hemorragia intraplaca, fissuras, erosões ou trombose^19^. Uma atualização dessa classificação detalhou as lesões complicadas (Figura 2), incluindo o tipo VII, lesões ateroscleróticas em que predomina a calcificação, e o tipo VIII, em que há predominância da fibrose^20^.

**Figura 1:** Corte histológico de feixe vascular periférico do membro inferior com lesão aterosclerótica: **A)** Feixe vascular periférico com obstrução completa da luz arterial. **B)** No detalhe, observa-se: mais perifericamente, a calcificação da camada média de Monckeberg (seta externa) e, na porção central, trombose oclusiva arterial em organização (seta interna).

**Figura 2:** Cortes histológicos de segmentos arteriais periféricos de membros inferiores com lesões ateroscleróticas. **A)** Lesão aterosclerótica do tipo fibroateroma (seta). **B)** Lesão aterosclerótica complicada com presença de calcificação na placa (seta).

A progressão das lesões não se dá necessariamente em uma única direção nem de maneira estritamente sequencial^20^. Ateromas, ou lesões avançadas do tipo IV, podem evoluir diretamente para os tipos V, VII, VII ou VIII, sem progredir necessariamente por cada etapa^20^. Outras classificações foram propostas na literatura, também a partir das lesões ateroscleróticas em artérias coronárias, considerando a presença de erosão e de rotura da placa, bem como a presença de trombos^18^.

A morfologia das placas ateroscleróticas tem papel importante na apresentação clínica. Enquanto as lesões obstrutivas crônicas clinicamente evidentes são representadas por fibroateromas, nos quais o núcleo lipídico é pequeno e a capa fibrosa é bem desenvolvida, o que torna a artéria mais rígida, as obstruções agudas por trombose ocorrem mais frequentemente sobre placas com amplo núcleo lipídico e capa fibrosa delgada ou inexistente, chamadas de fibroateroma de capa delgada, placa mole ou placa vulnerável^21^. O núcleo lipídico bem desenvolvido representa o predomínio do ambiente pró-inflamatório com numerosos macrófagos ativados secretando metaloproteinases que lisam a capa fibrosa e tornam essa placa mais predisposta a fissuras e ulcerações^9^.

A apresentação clínica pode variar na dependência do grau de obstrução do lúmen e da velocidade de desenvolvimento da obstrução. A obstrução crônica pode ser clinicamente evidente quando há redução da luz em pelo menos 70%. Contudo, as obstruções agudas, associadas à trombose, podem ocorrer sobre placas clinicamente silenciosas.

Nas artérias periféricas, uma pesquisa entre portadores de doença arterial obstrutiva periférica (DAOP) mostrou que, em segmentos arteriais de pacientes que sofreram uma amputação, existiam lesões ateroscleróticas avançadas, majoritariamente dos tipos V e VI, com grau de obstrução acima de 75% (nível 3b)^22^. Outro estudo, também com artérias de membros inferiores amputados por isquemia crítica, encontrou predominância de lesões do tipo fibroateroma e placas com fibrocalcificação, no segmento fêmoropoplíteo, e uma elevada frequência de trombos e calcificação da camada média nas artérias infrapoplíteas (nível 3b)^23^.

## ARTERIOLOSCLEROSE

As arteríolas fazem parte do leito microvascular, localizando-se entre as artérias terminais e os capilares, sendo responsáveis pela maior parte da resistência periférica ao fluxo sanguíneo^24^. As artérias terminais e as arteríolas controlam a resistência ao fluxo sanguíneo, contribuindo no controle da pressão arterial e regulando a perfusão tecidual^24^. A parede arteriolar possui componentes celulares e extracelulares, sendo a maior parte composta por células musculares lisas, que controlam o diâmetro do vaso, respondendo através da vasoconstricção ou vasodilatação aos diversos estímulos fisiológicos^5^^,^^24^.

A arteriolosclerose é o espessamento da parede arteriolar, o qual pode estar presente em diferentes tecidos e órgãos, sendo citado em diversas doenças. O espessamento das arteríolas já foi descrito entre os achados histológicos da hepatopatia esquistossomótica^25^. A HAS, um dos fatores de risco implicados na AT, também está relacionado à arteriolosclerose^26^^,^^27^.

Existem dois tipos de espessamento arteriolar: a arteriolosclerose hialina e a arteriolosclerose hiperplásica^7^. A arteriolosclerose hialina é frequentemente associada à HAS (nível 2b)^26^^-^28. O rim e o encéfalo são dois órgãos que poder ser comprometidos pela arteriolosclerose^26^^,^^29^. No rim do hipertenso, a arteriolosclerose hialina da arteríola aferente glomerular determina isquemia crônica e consequente glomeruloesclerose, responsável pela evolução para quadros de insuficiência renal crônica^30^. No cérebro, a arteriolosclerose hialina e a formação dos microaneurismas de Charcot-Bouchard são responsáveis pelos quadros de hemorragia intraparenquimatosa, importante complicação dos pacientes hipertensos. A arteriolosclerose hiperplásica, representada pelo espessamento laminar e concêntrico com redução importante do lúmen arteriolar, consiste em células musculares lisas com membranas basais espessadas e reduplicadas, associada a quadros de HAS grave^30^.

Mecanismos que regulam as atividades das arteríolas, a nível molecular, vêm sendo estudados em modelos animais experimentais^31^. Um estudo realizado a partir de dados de pacientes com doença de Alzheimer encontrou que a arteriolosclerose cerebral esteve associada a alterações cognitivas em idosos (nível 2b)^29^. Em membros inferiores de pacientes com DAOP avançada, uma pesquisa mostrou espessamento arteriolar em metade dos pacientes não diabéticos e em 63% dos diabéticos, sem diferença significativa entre os grupos (nível 3b)^22^.

## ESCLEROSE CALCIFICANTE DA MÉDIA DE MONCKEBERG

A ECMM também é conhecida por outras denominações, como calcificação da camada média arterial ou *medial arterial calcification* (MAC), arteriosclerose de Monckeberg ou *Monckeberg medial calcinose* 6^,^^32^ (Figura 3). Foi primeiramente descrita em 1903, como uma calcificação localizada na túnica média arterial, não envolvendo a íntima^32^^-^34. Considerando a descrição original, existe controvérsia na literatura se a ECMM inclui ou não as calcificações localizadas na lâmina elástica interna^7^^,^^32^. Um estudo observacional sugere que, na ECMM, a calcificação pode estar presente tanto na camada média arterial como na lâmina elástica interna (nível 4)^33^. Para tornar mais claras as características de cada lesão, alguns autores propõem uma mudança na classificação e na nomenclatura das lesões da parede arterial^7^.

**Figura 3:** Corte histológico de segmento arterial periférico do membro inferior com imagem de calcificação localizada na camada média arterial ou esclerose calcificante da média de Monckeberg (setas).

Diferentes mecanismos podem estar implicados na ECMM, como a ativação e migração de miofibroblastos a partir da túnica adventícia e a diferenciação de células musculares lisas^6^. Entre as distintas condições clínicas associadas à ECMM estão DM, doença renal crônica (DRC) e idade avançada^6^^,^^32^^,^^35^^,^^36^. De maneira geral, a ECMM é rara antes dos 50 anos de idade, mas pode ser mais precoce na DRC, ocorrendo mesmo na ausência de lesões ateroscleróticas^7^^,^^32^. A ECMM é considerada um dos tipos de calcificação vascular (CV) e vem sendo associada a aumento da morbimortalidade cardiovascular^6^^,^^35^. Um estudo de coorte, que incluiu adultos sem histórico de DCV, sugeriu uma possível associação entre o índice tornozelo-braquial (ITB) elevado (≥ 1,4) e um risco aumentado de mortalidade cardiovascular (nível 2b)^37^.

O ITB é um método diagnóstico não invasivo que, quando aberrante, sugere a presença de ECMM, podendo estar falsamente elevado em pacientes diabéticos com isquemia de membros inferiores^35^^,^^36^^,^^38^. A calcificação da camada média nas artérias distais (Figura 4) pode dificultar a compressão pelo esfigmomanômetro, resultando em um valor do ITB falsamente elevado. Estudo observacional encontrou que 11% dos pacientes diabéticos com diagnóstico de isquemia crítica apresentavam um ITB falsamente elevado (nível 3b)^38^.

**Figura 4:** **A e B)** Arteriografia digital com contraste evidenciando imagens de obstruções arteriais compatíveis com aterosclerose (setas). **C e D)** Radioscopia e radiografia sem contraste com imagens compatíveis com esclerose calcificante da média de Monckeberg (setas).

Classicamente, a ECMM não representa uma lesão obstrutiva, não comprometendo o lúmen arterial, mas pode coexistir com placas ateroscleróticas^7^^,^^39^. Mesmo que, diferentemente da lesão aterosclerótica avançada, a ECMM não tenha uma característica obstrutiva, ambos os tipos de CV podem levar a dificuldades intraoperatórias nas cirurgias arteriais^35^.

## CALCIFICAÇÃO VASCULAR

O termo CV é utilizado na literatura para denominar um conjunto de afecções caracterizadas pela calcificação ectópica em território vascular^6^. A ECMM, a calcificação na lesão ou placa aterosclerótica, a calcificação de válvulas cardíacas e a calcifilaxia são as quatro distintas moléstias incluídas nas calcificações vasculares^6^^,^^32^. Entre as doenças discutidas aqui, a calcificação pode estar presente nas lesões ateroscleróticas avançadas e na ECMM, podendo ser difícil distinguir o sítio exato da calcificação no exame tomográfico, se a íntima ou a camada média arterial^7^^,^^35^^,^^39^.

A tomografia computadorizada é um método de imagem que vem sendo empregado na formulação de escores de CV, em diferentes territórios arteriais, coronarianos, cerebrais e periféricos^40^^-^42. Um estudo prospectivo multicêntrico que avaliou a acurácia da angiotomografia computadorizada *multislice* de 64 canais, no diagnóstico das estenoses coronarianas, encontrou uma sensibilidade de 95% para estenoses ≥ 50% e de 94% para as estenoses ≥ 70% (nível 1c)^43^.

Nas artérias intracranianas, um estudo de coorte revelou uma associação entre os escores de calcificação tomográficos, a ocorrência de eventos vasculares, coronarianos, cerebrais ou periféricos e óbitos em pacientes com antecedente de acidente vascular cerebral isquêmico (nível 2b)^42^. Um estudo de coorte multicêntrico revelou que indivíduos com maior mensuração de cálcio em artérias coronárias apresentaram maior risco para eventos cardiovasculares por doença aterosclerótica, nos diferentes territórios, independente de idade, sexo e etnia (nível 2b)^44^.

Nas artérias periféricas, também vêm sendo propostos escores de CV para pacientes com DVP dos membros inferiores^35^^,^^40^. Um estudo observacional retrospectivo, que incluiu doentes com DVP sintomática, mostrou que diabéticos e portadores de DRC apresentaram maiores escores de calcificação e que o escore tomográfico de calcificação das artérias periféricas esteve associado a uma maior morbimortalidade cardiovascular (nível 2c)^40^.

A ECMM e a lesão aterosclerótica calcificada possuem fatores de risco em comum, como idade avançada e DM, podendo ocorrer isoladamente ou coexistir na parede arterial^39^. Ambos os tipos de CV podem dificultar o acesso, a cateterização, a angioplastia, as técnicas de reentrada, o clampeamento e a anastomose, tanto nas cirurgias convencionais como nos procedimentos endovasculares. Nas artérias infrainguinais, a CV representa um desafio às tentativas de recanalização, havendo o risco de perfuração, dissecção e embolização distal^35^. A literatura tem estudado as diferentes formas de CV e buscado alternativas de tratamento, quer seja prevenindo a calcificação, quer seja propondo novos dispositivos que permitam abordar as artérias calcificadas com melhores resultados^35^^,^^45^^,^^46^.

## CONCLUSÕES

Na prática médica, são utilizados termos semelhantes para descrever lesões arteriais distintas, o que pode dificultar sua compreensão. Esta revisão propôs discutir os conceitos implicados nesses diferentes termos.

Enquanto o termo arteriosclerose abarca todas as lesões que levam ao enrijecimento arterial, a aterosclerose compreende a presença de placas ateromatosas, com acúmulo de lipídeos na camada íntima arterial. A palavra arteriolosclerose implica no espessamento, celular ou hialino, de estruturas do leito microvascular, as arteríolas. Já a ECMM designa a presença de calcificação na lâmina elástica interna ou na túnica média de artérias musculares. As lesões ateroscleróticas e a ECMM estão incluídas nas chamadas calcificações vasculares, que vêm sendo estudadas como fatores de risco para eventos cardiovasculares.
